# Economic Evaluation and Transferability of Physical Activity Programmes in Primary Prevention: A Systematic Review

**DOI:** 10.3390/ijerph7041622

**Published:** 2010-04-09

**Authors:** Silke B. Wolfenstetter, Christina M. Wenig

**Affiliations:** 1 Helmholtz Zentrum München, German Research Center for Environmental Health, Institute of Health Economics and Health Care Management, Ingolstädter Landstraße 1, 85764 Neuherberg, Germany; E-Mail: wenig@bwl.lmu.de; 2 Ludwig-Maximilians-Universität München, Institute of Health Economics and Health Care Management and Munich Center of Health Sciences, Ludwigstr. 28 RG, 80539 München, Germany

**Keywords:** economic evaluation, cost-effectiveness, physical activity intervention, transferability, cost-utility, primary prevention

## Abstract

This systematic review aims to assess the characteristics of, and the clinical and economic evidence provided by, economic evaluations of primary preventive physical exercise interventions, and to analyse their transferability to Germany using recommended checklists. Fifteen economic evaluations from seven different countries met eligibility criteria, with seven of the fifteen providing high economic evidence in the special country context. Most of the identified studies conclude that the investigated intervention provide good value for money compared with alternatives. However, this review shows a high variability of the costing methods between the studies, which limits comparability, generalisability and transferability of the results.

## 1. Introduction

One of the WHO European regional targets for the twenty-first century is that ‘by the year 2015, people across society should have adopted healthier patterns of living’ [1]. The WHO target refers to a variety of key behavioural factors contributing to the large societal burden of disease, one of them being physical inactivity. The global estimate for the prevalence of physical inactivity among adults is 17% [2]. Overweight and inactivity have been shown to be associated with several risk factors such as hypertension, diabetes mellitus type 2 and dyslipoproteinemia [3].

Increased physical activity and dietary intervention have been shown to have a preventive effect and/or a strong effect on health risks, e.g., effecting a reduction of cardiovascular risk factors [3,4]. Many reviews and meta-analyses confirm this positive correlation between physical activity and psychological, physiological and social effects focussing on secondary prevention [5–12]. Although the literature strongly confirms the effectiveness of physical activity interventions, translation of these findings into public health practice has been limited. There is a need for data on the cost-effectiveness of physical exercise intervention programmes to support policy-makers in decisions based on valid information about the value of those interventions.

There exist many reviews of cost-effectiveness of secondary prevention programmes that include physical exercise as one treatment option, e.g., [13–17]. Earlier reviews examine economic results of primary and secondary preventive physical activity programmes [15,18–20]. Gordon *et al.* analysed the evidence for cost-effectiveness of health behaviour interventions that comprise the major behavioural risk factors for chronic diseases including smoking, physical inactivity, poor diet and alcohol abuse. The reported incremental cost-effectiveness ratios (ICER) per life years gained (LYG) range from €1,800 to €47,500, and the costs per quality adjusted life year (QALY) vary between €2,200 and €53,500 (2006 Euros) for physical activity interventions. In comparison, ICERs for smoking cessation are consistently low, e.g., <€14,000 per LYG. Gordon *et al.* did not differentiate between primary and secondary prevention [18]. Hill *et al.* analysed the effect that economic factors such as industry practices have on eating and physical behaviours, and Hagberg and Lindholm focussed on different aspects of outcomes and equity in health [15,19]. NICE also included secondary prevention in its rapid review of the economic evidence, and Shepard examined the economics of fitness with special focus on worksite programmes and found an immediate return of CAD2-8 per dollar invested [20,21].

Systematic reviews can help making well-informed decisions on which intervention to adopt for a particular country or region. For maximum usefulness, cost-effectiveness studies should be transparent, of high methodological quality and high economic evidence. The aim of this systematic review is to analyse the clinical and economic evidence in the specific country context provided by economic evaluations of primary preventive physical activity programmes, and to discuss the transferability of the findings of these studies to Germany.

## 2. Methods

### 2.1. Search Process

In order to identify all relevant studies published before December 2009, the database PubMed/Medline was searched for all possible combinations out of three groups of terms. The first group included different terms assigned to physical activity, such as ‘Movement’ OR ‘Exercise’ OR ‘Exercise Therapy’ OR ‘Exercise Test’ OR ‘Exercise Movement Techniques’ OR ‘Exercise Tolerance’. The second group broadly described different methods of economic evaluation: ‘Costs and Cost Analysis’ OR ‘Economics’. The third group contained terms for prevention: ‘Prevention and Control’ OR ‘Primary Prevention’ OR ‘Accident Prevention’ OR ‘Health Promotion’ OR ‘Centres for Disease Control and Prevention (U.S.)’. Most of the selected MeSH-terms are generic terms, each encompassing a set of subordinate search words. Thus, for example, the search for ‘Cost-Benefit Analysis’ is covered by the search for ‘Costs and Cost Analysis’ [MeSH]. Similarly, ‘motor/physical activity’ is assigned to the MeSH-term ‘movement’. Additional searches on the DIMDI, EconLit and Embase databases were carried out analogously. Based on assessments of the abstracts, a list of relevant papers was derived. Papers were deemed potentially relevant if outcomes and costs of a primary prevention physical activity programme were evaluated.

### 2.2. Inclusion and Exclusion Criteria

Only studies published in peer-reviewed scientific journals in English, Dutch, French and German language before December 2009 were selected for this review. This review is limited to trial-based economic evaluations of primary prevention programmes focussing on an adult population. This type of study with strong and convincing evidence for efficacy is of high priority to the German Institute for Quality and Efficiency in Health Care (IQWiG) [22]. Therefore, studies that were based on secondary research, literature-based modelling and literature reviews were excluded. Reported findings were not included if they were anecdotal and/or not evaluated. The present review is limited to full economic evaluations (primarily cost-effectiveness, cost-utility and cost-benefit analyses) that report the cost-effectiveness of primary prevention programmes based on physical exercise.

### 2.3. Data Extraction and Criteria

**Figure 1.**
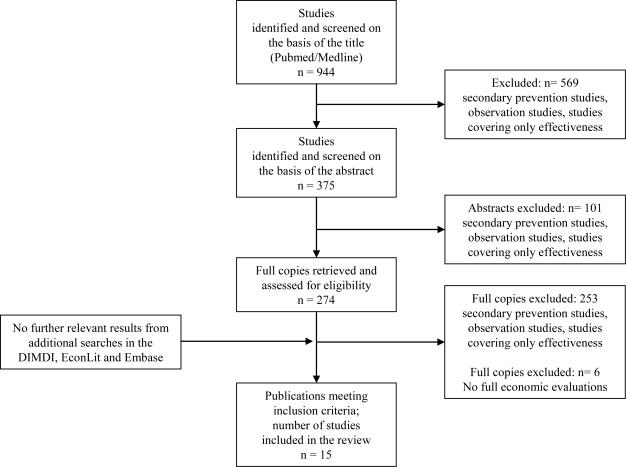
Flow of information through the different phases of the systematic review according to PRISMA guidelines [23].

In total, 944 studies were identified from the first search in PubMed, including all studies that were completed before December 2009. Many of these were secondary prevention studies, observation studies, or studies that covered only effectiveness. Others were reviews, focussed on children or were not peer-reviewed, and, thus, were excluded from further examination. On the basis of the title, 375 papers were considered as relevant for the review and were obtained in abstract. One hundred and one abstracts were excluded, the remaining 274 articles were examined with regard to the inclusion and exclusion criteria. In total, 15 of the finally selected primary research studies described an economic evaluation of primary preventive physical activity programmes for adults. Additional searches in the DIMDI, EconLit and Embase databases showed no further relevant results. Following the recommendations of Moher *et al.* Figure 1 (Appendix) describes the flow of information through the different phases of the systematic review [23]. Data extraction and assessment were undertaken and checked by two researchers.

### 2.4. Study Characteristics and Key Findings

All 15 studies were briefly described by means of important characterising aspects, including ‘type of physical exercise intervention/alternative/length of intervention, outcomes, study population, country/setting/year(s) of the study, study design/type of economic evaluation, economic key findings and clinical/economic evidence’ (Table 1). In addition to presenting the key findings in their local currency and price year, costs were converted to Euros using purchasing power parities (PPP) [24] in order to facilitate comparisons across studies. These results were then inflated to 2008 prices using general price indices (GDP) [25]. If information on the base year for prices was missing, the year of the intervention was used instead, if indicated.

### 2.5. Clinical and Economic Evidence

The level of evidence regarding the effectiveness of the intervention was evaluated using the evidence gradients developed in evidence-based medicine [22,26–28]. Therefore, all selected studies were assessed against relevant criteria such as randomisation, blinding and concealment of allocation, comparability of the groups, description of the drop-out rate or compliance/participation rate and the intention-to-treat approach [28–30].

Different internationally recommended guidelines for economic evidence of trials already exist [28,31–34]. The CHEC-list (Consensus on Health Economic Criteria) by Evers and colleagues [32], which is based on expert consensus of 23 international experts in a DELPHI-panel, seemed to be most suitable for this review. Each criterion has to give insight into the quality of the study performed, rather than into how the study is performed. The CHEC-list is suitable for systematic reviews, which include full economic evaluations based on clinical trials, e.g., case-control studies, cohort studies or randomised controlled clinical trials, which compare two or more alternatives, and in which both costs and outcomes of alternatives are determined aimed at more transparency and comparability [32]. The CHEC-list provides 19 yes-or-no questions, one for each criterion of evaluation. If the information pertinent to the question is not available in the reviewed article, the assessor ticks it with ‘no’; if the question has been fully answered in the article, the statement would be ‘yes’. In this review, the CHEC-list is applied in a modified manner. When a criterion in an article could be identified but not fully answered, the evaluation was 0.5. The statement ‘no’ was valued with 0 and ‘yes’ with 1. An overview of the detailed evaluation of the economic evidence against the CHEC-list can be found in Table 2 in the appendix.

### 2.6. Transferability

A prerequisite for examining the transferability of international studies to Germany requires that methods, data resources and study results are transparently and comprehensible described [22,26,35]. Welte *et al.* systematically identified the factors that may influence the transferability of health economic study results between countries. These transferability factors can be differentiated into three categories: methodological characteristics (perspective, discount rate, medical cost approach, productivity cost approach), healthcare system characteristics (absolute and relative prices in health care, practice variation, technology availability) and population characteristics (disease incidence/prevalence, case-mix, life expectancy, health status preferences, acceptance, compliance, incentives to patients, productivity and work-loss time, and disease spread). All potential transferability factors have to possess four characteristics: influence on outcomes of economic evaluations, international variation, measurability and being distinguishable from other factors [35]. Aspects of the transferability of the identified studies are discussed in detail.

## 3. Results

### 3.1. Study Characteristics and Key Findings

Altogether, 15 economic evaluations of physical activity programmes in primary prevention were identified. All were published in English between the years 1992 and 2008. The study characteristics of all economic evaluations, including keyfindings and methodological issues can be found in Table 1.

The *type of physical exercise programme* varied strongly, e.g., a group-based community programme or an individually supervised fitness programme. Personal supervised or written unsupervised advice via email, postal way or telephone calls was provided.

The adult *study populations* of the reviewed interventions ranged over different age groups and characteristics, including, e.g., employees, patients of general practitioners or uninsured women with low income. The interventions included a range of targets (e.g., from moderate to high impact exercise) and delivery methods (e.g., general practitioner, health professional advice, home–based programmes led by nurses or physiotherapists).

The optimal *time horizon* of an intervention depends on the evaluation of all outcomes, *i.e.*, the time horizon is not long enough if outcomes ensue after the evaluation period. The length of the reviewed interventions ranged from 10 weeks to 12 years. The follow-up time in the studies reviewed ranged from no follow-up to as long as 12 years [36,37].

The *outcomes* were selected according to the respective study aim and therefore varied from specific measures, e.g., activity change, work participation or deleterious health events (falls) to generic measures, like QALYs or DALYs. Outcomes were valued and calculated with different scores and indexes derived from literature or statistics, e.g., the health state utility index by Brazier [38,39] or a 10 year CVD risk score for angina pectoris, myocardial infarction, heart failure or CVD mortality [40].

Regarding the *clinical effectiveness*, the reviewed interventions were significantly more effective than alternative interventions or usual care in twelve studies, including increase in total expenditure of energy [38,41,42], in quality of life [36–39,43,44], in DALYs [45], in VO_2_(max) [46,47] and reduction in falls [48–50]. No significant differences were measured in lower absenteeism rates, less healthcare claim reimbursement [37], decrease in 10 year probability of CHD [40], positive changes in mortality rate, survival times, or admissions to hospital [39] and an increase of occasions of physical activity [42]. High-level intervention is marginally more effective in improving diet, physical fitness and blood cholesterol [37,51].

The authors of the analysed studies used different *types of economic evaluations* influencing the *economic key findings* presented. Twelve of the identified studies performed a cost-effectiveness analysis. The cost-effectiveness studies of Robertson *et al.* were all included, even though they assessed three similar home-based programmes for elderly people aimed to prevent falls. Age and sex of the target populations differed between the studies as well as the delivery of the exercise programme (by district nurse, general practice nurse or physiotherapist). The results show that the costs per fall prevented are lowest in the study including women over 80 years trained by physiotherapists (NZD314/ €261 after one year and NZD265/ €220 after two years) [48] compared with mixed study populations aged over 80 years (NZD1,519/ €1,202) trained by a general practice nurse [49] and a study population aged over 75 years (NZD1,803/ €1,423) trained by a district nurse [50].

The healthcare cost savings due to an employee fitness programme are calculated to be around NZD1,756 (€1,268) [38] per person converted from a sedentary lifestyle to an active state. As compared with the costs of AUD69 (€62) per patient to become more active, calculated by Sims *et al.*, this result seems rather high [45]. Elley and colleagues only implemented a cost-effectiveness analysis, even though they assessed the parameters of the SF-36, a QALY index instrument [38]. The results of these outcomes were not used for a cost-utility analysis. Two studies reported net savings due to physical intervention [37,41].

Shepard and colleagues conducted cost-effectiveness and cost-benefit analyses of the 10 year follow-up of an employee fitness and lifestyle programme [37]. They calculated a return on investment of CAD6.85 (€7.64) over 12 years. Prior studies on this programme were excluded from this analysis because of an incomplete economic evaluation method [52,53]. The worksite physical activity counselling programme analysed by Proper *et al.* resulted in total net costs of €305 during the intervention, but €635 benefits from sick leave reduction after one year in favour of the intervention [41]. In comparison, programme benefits per worker per year of another employee fitness programme were estimated to be CAD679 (€757) [37].

Four of the identified studies performed a cost-utility analysis based on QALYs [36,39,43,44]. The incremental cost per QALY varies between NZD 2,053 (€1,483) [44] and €18,363 [39]. Sims *et al.* calculate a rate of AUD3,647 (€3,258) per DALY saved for the delivery of effective advice on physical activity by GPs to patients [45]. The results of the analysed studies are difficult to compare, first due to the differences of the physical activity programmes and their aims, second due to methodological differences especially the high variation of outcomes and third, the lack of transparency of the methods and data used. Table 1 provides an overview of all economic key findings.

### 3.2. Clinical and Economic Evidence in the Special Country Context

The evidence regarding the effectiveness of an intervention depends on the study design and the bias, which can influence results of a study because of a systematic error, deviation in results or inferences from the truth [22,26–28]. The level of evidence on effectiveness depends on the study design, with the highest ranking given to randomised clinical trials, which were conducted in 13 studies. Only two studies conducted a controlled trial without randomisation [37,49].

It is unclear whether the studies incorporated blind or concealed trials. An intention-to-treat analysis was conducted in seven studies [38,42,46,48–51]. All studies, at least briefly, mentioned the compliance and participation rates of their study populations.

A potential selection bias in physical exercise programmes cannot be fully excluded, e.g., whether the control group also takes part or only the motivated people take part in the exercise trial as in the study by Elley *et al.* [38]. Dzator *et al.* stated over-representation of higher economic status in their study population [51], and Munro excluded those with a physical activity score in the top 20% assuming little gain from additional exercise there [39]. Stevens *et al.* discussed the limitation of quality and transferability of their results because of the possibility of an instructor bias [42]. The results of three other physical exercise programmes for older people by Robertson *et al.* were possibly influenced by an instructor bias, e.g., by one motivated research physiotherapist [48] or by an educated district or general practice nurse [49,50]. Finkelstein *et al.* did not discuss the baseline comparability of the two intervention groups [40]. Overall, seven RCTs had a low or very low risk of bias, showing high clinical evidence [41–43,47,48,50,51], and six RCTs had a high risk of bias, thus moderate clinical evidence [36,38–40,44,45]. There were only two case-control studies, one with a low [49] and one with a high risk of bias [37].

Next to the clinical evidence, the economic evidence in the special country context is important for decision-makers. In the following, the main methodological weaknesses affecting the level of economic evidence will be summarised for each study. The detailed evaluation of the economic evidence and factors affecting this evaluation are presented in the Appendix, Table 2.

The three studies by Robertson *et al.* are of high economic evidence. They included implementation costs as well as overhead costs of each hospital item and excluded the government goods and consumption taxes [48–50]. However, ethical and distributional issues are not discussed appropriately; these were only commented on in three studies [36,37,40]. The missing discount rate in one study with a 2 year time horizon should have been discussed, but the impact on the results would have been rather small.

The economic evidence of the three identified lifetime models is medium to high, but the methods applied for measurement and valuation of costs should be described in more detail [43–45]. Dalziel *et al.* used a health system perspective only and a model based on many assumptions [44]. The methods of discounting, as well as the modelling approach, were not clearly described by Sims *et al.* [45].

The two cost-utility analyses included are of moderate to high economic evidence. However, the time horizon of the intervention by Chen *et al.* was 12 weeks and only programme costs were assessed, whereas healthcare utilisation was not monetised. In addition, the cost measurement in physical units was not clearly described and the sensitivity of the results to changes in uncertain values was not analysed [36]. Furthermore, the perspective was not clearly stated. Similar to Robertson *et al.*, Munro *et al.* did not discount costs and effects in a 2 year intervention; moreover, they used a health service perspective only [39]. The economic evidence of the study by Dzator *et al.* is limited due to the short time horizon of the intervention; none of the perspective, the physical units for cost calculation or the year of intervention were stated clearly [51]. The analysis by Elley *et al.* has only minor economic limitations, and, therefore, a high economic evidence. However, only the costs were discounted. The study by Finkelstein *et al.* has different limitations, as there was no sensitivity analysis calculated, and the perspective, the unit costs and the price year were not mentioned. Moreover, only effects were discounted [40]. Proper *et al.* conducted an economic evaluation of moderate economic evidence, as the price year and physical units were not stated. Additionally, the time horizon of the intervention including the follow-up was only 9 months and costs were calculated from a municipal service rather than a societal perspective [41]. Although the study of Shepard *et al.* has the longest time horizon of 12 years, the economic evidence is rather limited, as the physical unit costs, study years, ICER and perspective were not explicitly stated. Moreover, there was no sensitivity analysis and future costs and outcomes were not discounted [37]. The cost-effectiveness analysis of Stevens *et al.* was categorised as moderate economic evidence. The perspective, the physical units, ICER and year of intervention were not mentioned. Furthermore, the valuation of costs and some relevant cost components were not reported [42]. According to the criteria of economic evidence stated above, the study of the Writing Group showed the highest limitations. The focus was on reporting of the effects of the intervention, whereas the economic methodology of the ICER calculation was not described. Also, there is no indication for discounting of future costs and effects [47].

The most important methodological issues are summarised in the following. In general, the collection of costs depends on the chosen perspective. As far as the perspective of the study was stated, it varied between the healthcare payer’s perspective [38,39,43–45], the company’s viewpoint [41] and the recommended societal perspective [38,43,48–50]. The methodological quality of costing varied among the studies; five of the 15 studies identified valued the costs appropriately. For a complete economic evaluation not only programme costs, but also cost savings due to the health effect of the programme, are of special importance. Savings include direct medical costs, direct non-medical costs and indirect costs due to utilisation of healthcare services. Indirect costs induced by productivity losses will only be included if a societal or company perspective is chosen.

The cost data collection depends on the study type. All direct medical costs were assessed either by questionnaire [41], statistical indices [38,39,41], healthcare insurance administration records [37,39,50], postcard calendars [48–50] and/or by telephone [48,50]. The valuation of resource consumption varied between market prices and charges. Except for Robertson *et al.* [48–50], all other economic evaluations did not mention overhead costs or the valuation of the costs. All costs were declared in their own country’s currency.

A separate and transparent presentation of how the quantities of resource use were determined was found in only five of the 15 studies [38,39,48–50]. An incremental analysis was performed in 13 of the 15 studies. Outcomes were identified, measured and valued appropriately in 13 of the 15 studies. The practice of discounting in economic evaluations of healthcare interventions was analysed by Smith and Gravelle. The authors recommended the need for discounting if the evaluation takes more than 18 months [54]. Regarding the studies with a longer time horizon, only the three model-based evaluations included discounts [43–45]. Hence, the valuation of the reviewed studies without discounting was calculated as 1 if the evaluation period was less than 18 months and 0 if the time horizon was more than 18 months. A broad range of important but uncertain variables was investigated in sensitivity analyses in 13 of the 15 studies, e.g., completion rates of participants [51], session fee of exercise leaders, number of attendees per session or different approaches to calculating costs per QALY [39].

In summary, seven studies fulfilled high standards of methodological quality, with more than 80% of quality items meeting the criteria [38,39,43,45,48–50]. Six economic evaluations were of moderate quality, with 51–80% of quality items meeting the criteria [36,40–42,44,51]. Two studies demonstrated poor quality standards, fulfilling only 50% or less of the quality criteria assessed [37,47].

### 3.3. Transferability

Transferability can be interpreted in a national context in terms of transferability of the results to another region or setting, or in an international context. Geographic transferability of the results of economic evaluations of physical activity programmes from one country to another has the potential to make a more efficient use of national and international evaluations because the implementation of primary clinical trials is very expensive and time-consuming. However, inappropriate transfer of economic data can provide misleading results and can lead to an inefficient use of scarce health resources.

Before the transferability to the German context can be examined, three questions must be answered positively. First: Is the considered technology comparable with a technology used in Germany? Second: Is the comparable intervention relevant for Germany? And third: Is the quality of the study acceptable for Germany, and does it fulfil international methodological standards? Transferability of the intervention is not given if one question is answered with ‘no’ [22]. The considered technology here, different types of physical activity interventions, is comparable with German physical activity programmes and is also relevant for the German context, because of a high prevalence of physical inactivity. Therefore, the first two questions can be answered with ‘yes’. However, it is not apparent whether German companies are comparable with American or Canadian companies and their employee fitness activities and fitness centres [37,41]. But the need for health promotion and physical activity interventions for sedentary people has also been stated in Germany [55,56]. The third question can also be answered in the affirmative, even if the clinical and economic evidence provided by the studies varies (Table 1).

Assessing the transferability of economic evaluation results of physical activity programmes is a complex and difficult task. The transferability of the identified studies will be discussed according to the checklist of Welte *et al.* [35]. The criteria of transferability are partly similar to the criteria of high economic evidence discussed above. To avoid overlapping, only those transferability criteria that differ from the ones above will be discussed in detail in the following section.

The methodological characteristics include the perspective, the discount rate, the medical cost approach and the productivity cost approach. The perspective of a considered study is very important in terms of transferability to the German healthcare context. The IQWiG proposes the perspective of the statutory health insurance; this proposal is controversial [22,26]. The societal perspective is more accepted and preferred [57]. First, sectors other than the health service may incur costs or benefits as a result of healthcare interventions and the societal perspective can help detect cost shifting between sectors. Second, a narrow perspective takes no account of alternative uses of resources outside the healthcare sector, which may yield greater welfare to society. The concept of opportunity cost reflects this broad concern for society’s total welfare [58,59]. Therefore, with respect to the perspective chosen, six studies can be considered as being transferable [38,48–50].

The Hanover Consensus has proposed a discount rate of 5% for outcomes and costs for Germany [57]. Only three studies used a discount rate of 5% [38,44,51], and three studies used 3% [40,43,45].

The medical cost approach implies different costing methods for direct medical costs. They can be calculated with charges, fees, per diem costs and real market prices, as well as different levels of aggregation of resources. Overhead and capital costs, which can be measured in different ways, may be included [60]. The price year is important in terms of transferability, as well as detailed cost measurement and valuation. Charges and fees, such as costs of a general practitioner visit depend on the type of healthcare system and the healthcare provider. For costing of healthcare services most of the studies used official charges, like Elley *et al.* [38,39,50].

Some studies valued units with fair market prices in the currency of their individual country in the year of assessment, e.g., Robertson 2001 [37,39–41,48–50]. Other studies valued their units of resource use by actual prices [51], used the consumer price index in the observation period [38] or did not explain their proceeding sufficiently, e.g., Shephard 1992 [37,42,47]. If the valuation of costs is not described in detail, the possibility of examining the transferability of results is very limited.

Due to missing information on the price year of adjustment, the transferability of the results of six studies is very limited [36,40–42,47,51].

Average labour costs or wages, the average productivity and the friction time can differ between countries. The average productivity is affected by parameters like labour force participation rate, unemployment rate and percentage of part-time workers. The friction method is used for calculating the indirect costs, and the length of the friction time can strongly influence the productivity cost estimate [35]. Three of the reviewed studies include indirect costs due to productivity losses [38,41,43]. Transferability of the indirect costs according to the study of Proper *et al.* is not possible because there was no assignment of physical units or price year of the follow-up period [41]. The study results of Elley *et al.* can be transferred with constraints because the average stoppage days in New Zealand were 1.9 days per employer in the year 2005 [38,61], whereas in Germany the average days of sick leave were stated as 10.4 [62,63]. For the calculation of indirect costs, the humancapital approach is the standard approach in most countries, except for the Netherlands where the friction cost method is proposed [64–66]. Two studies calculated the indirect costs based on sick leave days, leading to comparable results between the humancapital and the friction cost method [38,41]. Lindgren *et al.* included the indirect costs related to loss of production due to disease and costs in added years of life based on Swedish literature [43]. Even if the Swedish and German data were very different, parts of the model could easily be substituted by German data. Robertson *et al.* did not calculate the indirect costs because the target group was retired [48–50].

Concerning healthcare system characteristics, the most relevant factors are absolute and relative prices in healthcare, practice variation and technology availability [35]. Technology availability depends on the country-specific healthcare system. The reviewed studies were conducted in the Netherlands, the United States (US), Australia, New Zealand, the United Kingdom (UK), Taiwan, Sweden and Canada. Compared with Germany, these countries have rather different healthcare systems, including market structure and regulation, staff characteristics and effects of learning, and incentives to healthcare providers [67]. WHO recommends that high priority should be given to national policies in order to influence patterns of physical activity for an effective prevention of non-communicable diseases. Such policies should encompass broad measures involving different sectors such as health, agriculture, education, transportation, sport, industry, commerce and civil society [68]. The contribution-funded German healthcare system, separated into public health insurance and private insurance, shows limited similarities to the healthcare systems of the Netherlands and of Canada [37,41]. The results of the other studies, e.g., UK [39,42] or US [40,47] or Taiwan [36] cannot, or only with severe limitations, be transferred to Germany. Technology availability and costs may also vary between urban and suburban areas in a country, which can hamper the transferability of the results by Robertson *et al.* [48–50].

Absolute and relative prices can differ between countries and over time. Both prices can have an important impact on cost-effectiveness of technologies or treatment decisions. For physical activity programmes, the absolute and relative prices of programme costs can also be important for transferability. Programme costs were considered in 14 studies, but not all of them separated quantification and valuation of resource use [36–45,48–51]. Information on physical units is important for the transferability because prices such as the wage of a trainer can differ from one country to another. However, this is not reported properly in all studies.

Practice variation may influence the effectiveness of the intervention of different treatment practices, which can result in different outcomes. For example, Fretheim *et al.* described the patterns of antihypertensive drugs in ten countries and explored possible reasons for an inter-country variation. Suggested factors to explain inter-country variation included reimbursement policies, prescribing patterns, traditions, opinion leaders with conflicts of interests, domestic pharmaceutical production and clinical practice guidelines [69].

Eight reviewed studies were conducted in general practices in New Zealand [38,48–50], the UK [42], Australia [45] or the USA [47]. Their transferability of the results could be limited because of inter-country variations in charges, politics and traditions. The transferability of worksite physical activity programmes depends on how much an employer will benefit from reduced healthcare costs. Thus, the results of the Dutch intervention programme [41] may be transferable to Germany.

The transferability of results of an intervention also depends on the population characteristics of the specific target group. This includes the prevalence or incidence of inactivity, the case-mix, the life-expectancy of the population, the health status preferences, the compliance with and acceptance of the physical activity programme, and the valuation of productivity and work-loss time.

The prevalence of physical inactivity differs between countries, e.g., Germany with 35.6% during the years 1990–92, UK 9.0% in 1990, USA with 14.4% in 1992, Canada with 33.0% during the years 1981–88 and Australia with 17.5% during the years 1984–87 [70]. The prevalence of physical activity in Canada [37,52,53] is the most similar to the German situation.

The impact of physical inactivity on the population’s health can be measured by use of the PAF (disease-specific population attributable fractions) in the number of DALYs (disability-adjusted life-years) or deaths. This is the proportion of the disease burden of the population that would be eliminated if the current exposure to the risk factor were reduced to the feasible minimum. PAFs can influence savings and, therefore, the ICER of an intervention programme. WHO estimates that in the year 2002 the joint PAF for inactivity for Germany was about 3.2% of DALYs and 5.9% of number of deaths, for the Netherlands (DALYS: 2.7%; deaths: 4.5%) and for the UK (DALYS: 3.1%; deaths: 5.5%) [71]. The differences in PAFs for inactivity are between 0.5% of the DALYs and 1.0% of the deaths between these three countries. Hence, the transferability of the studies from the Netherlands and the UK [39,41] to Germany could be given. Life-expectancy from birth of the population of Australia, Canada, Germany, Taiwan, New Zealand, the UK, the Netherlands and USA ranges between 80.6 years of age (Australia) and 77.8 years of age (USA) in 2004 [72]. Therefore, the population of the reviewed studies have a comparable life expectancy and the results could be transferred to Germany.

The case-mix of the target group, *i.e.*, age, sex, race, education and other risk factors, may have a strong impact on the effectiveness of a physical activity programme, the participation, and the indivual utilisation of healthcare [73–75]. In terms of transferability, it is necessary that the target population fits to the transferred country. Reviewed physical activity programmes are different with regards to case-mix in terms of age groups, inclusion and exclusion criteria. For example, transferability of studies to Germany could be limited because of a very specific study population, e.g., uninsured, low-income women in the USA [40], other strong inclusion criteria or an unknown target group [37]. The transferability of the results is limited if an intervention is only effective for a very specific target group, such as for people older than 65 years or men aged 60 years [43,48–50]. These results can hardly be generalised and transferred beyond the population under research.

Between countries, preferences for different health states and trade-offs between lifetime and life quality can vary. Johnson *et al.* compared directly elicited valuations for EQ-5D health states between the US and UK general adult populations. The US mean scores were numerically higher than those for the UK for 39 health states. Greater differences in valuations were present only in health states characterised with extreme problems [76]. Other authors compared the Finnish and US-based visual analogue scale valuations of the EQ-5D measure, finding that country-specific differences were not large and not dependent on the dimension and the level of problem [77]. There is a lack of published data that have evaluated and compared the SF-36 health states and their preference weights for Germany, the UK and New Zealand. Four identified studies have assessed QALYs as an outcome parameter, conducted in the UK, Taiwan, New Zealand and as a sensitivity analysis also by Lindren *et al.* in Sweden [36,39,43,44]. The QALYs of two studies reviewed from UK and New Zealand are derived by using the SF-36 as it is used in Germany. These results of the different health states evaluated could be transferred due to the assumption of minimal differences in health states and quality of life [78–80].

Acceptance of physical activity programmes is an important precondition for the success of such a programme. Culture and religion are typical parameters that might influence acceptance. As some religions, for example, advocate the separation of men and women, a joint physical exercise for women and men would not be accepted in those countries. Fourteen of the reviewed studies were conducted in Western countries in their study population. Therefore, the transferability to Germany could be given.

Compliance and participation rate of a physical exercise programme influence the effectiveness. Hence, a better compliance could affect outcomes if the intervention is suitable for the target group. This important dimension was fully announced by 12 studies [36,37,39–43,45,47–50]. Differences in compliance with physical activity between countries were not published. All studies that mentioned compliance will be positively assessed with regard to transferability.

In terms of productivity and work-loss time, all seven countries are high-income countries [81] for which no relevant differences would be assumed.

In summary, none of the reviewed study results can be transferred without limitations. According to the criteria discussed above, five studies and their results cannot be transferred to the German setting at all because of, for example, unspecific outcomes, no declaration of physical units and price year, a very specific setting or perspective such as the viewpoint of a US company or a very specific target group [37,40,42,47,51]. One study met half of the eligibility criteria but the results cannot be transferred because of missing physical units, no price year and a company perspective [41]. Five studies can only be transferred to Germany with limitations, e.g., limitations due to different healthcare systems or because of missing price year [36,38,39,44,45]. The study of Elley *et al.* provides only weak evidence for its efficacy results because of various sources of bias as, for example, it cannot be excluded that only motivated people participated in the study [38]. Another study that could be transferred to Germany with moderate limitations is the physical activity programme of Munro *et al.*, which was conducted in a community of the UK from a health service perspective [39]. The three studies by Robertson *et al.* investigating programmes to prevent falls in elderly people might be transferable with minor limitations. Differences in health care system might affect costs, e.g., in terms of healthcare savings due to reduced utilisation of healthcare services. Furthermore the results can only be transferred to a similar type of service population [48–50]. The study by Lindgren *et al.* meets most of the criteria, and thus seems transferable with minor limitations. A positive aspect is that the model is based on the Framingham risk scores calculated for Germany and the UK. However, the intervention is not described properly, only includes men aged 60 years and does not allow for conclusions regarding other study populations [43]. Appendix Table 3 provides a summary of the major limitations affecting transferability.

## 4. Discussion

In summary, 15 economic evaluations of primary preventive physical activity programmes from seven different countries (New Zealand, the USA, UK, Canada, Australia, Taiwan, the Netherlands) were identified that met eligibility criteria. Most of the identified studies conclude that the investigated intervention provides good value for money compared with alternatives or even cost saving. However, it is not possible to rank these results. It is difficult to asses to what extent the variation in cost-effectiveness is due to different methods of valuing costs or cost savings and/or health outcomes. This review shows a lack of standardisation of what constitutes either costs or outcomes in such interventions and their evaluations; the inclusion of cost variables such as for gym, equipment, salaries of site health personnel are not standardised. The level of evidence on effectiveness depends on the study design, with the highest ranking being randomised clinical trials, which were conducted in 13 studies (including cluster randomised trials). According to the above stated criteria, seven studies were of high and eight studies of moderate clinical evidence. In total, seven studies provided high economic evidence in their special country context, with at least 80% of the quality criteria fulfilled. Six economic evaluations were deemed moderate and two poor in methodological quality with only half of the quality criteria met. Only four studies seem to be transferable with minor limitations, five studies may be transferable with moderate limitations and six studies are not transferable due to substantial constraints regarding the criteria of Welte *et al.* [35]. Those were, in general, poor in the ‘population characteristics’ and ‘methodological characteristics’. Very few details were given about the study sample and about the calculation of the cost results. It would be of utmost importance that authors present costs and physical units separately, provide the sources of the resource consumption data or unit prices, methods of valuation, including price year and discount rate, the perspective chosen and thus cost components included. These aspects were not stated in several studies, although they are essential for the economic evidence [32] and transferability of study results [35].

The main limitations of this review are that the collection of publications was limited to those referenced in given databases. Only English and German papers were included, missing all publications in other languages. The selection and analysis of the studies was conducted by only two researchers, leaving room for a possible bias, however the rate of concordance was around 90%. The costs of the studies were adjusted to Euros (2008) to show better comparability between the studies. But this explanatory power is limited because country-specific healthcare systems, their prices and charges, *etc.* were not taken into account in the calculation. In order to generalise the results to other settings, regions or countries, an economic re-analysis is recommended to account for different inactivity prevalence or incidence, healthcare systems, absolute and relative prices, *etc.* However, comprehensive and standardised checklists were used to establish the methodology, quality of evidence and transferability of each study. The level of overall economic evidence was assessed using a non-weighted quantitative score. The use of scales with summary scores to distinguish high and low quality studies is, however, highly controversial [82]. In this study, the score values are only used as support to get an estimate for overall quality. Nonetheless, the focus is clearly on the qualitative analysis of the single quality criteria, as this score does not account for the importance of the single criteria in a special context by weights.

In general, intervention programmes and the time horizons of their subsequent evaluations must be of sufficient duration to substantiate clinical effectiveness and/or cost outcomes. Results from a review by Pelletier suggest that a programme should be maintained for a minimum of around 3–9 months to show results in health risk reductions and/or cost-effectiveness [83]. Clearly, programme effects are more likely to be maintained if the programme is continued for a longer duration. Ideally, health promotion should be supported by decision-makers, politicians and stakeholders, so that they can become part of the setting or organisation. Physical exercise programmes of well-executed large-scale corporate initiatives can show that when such programmes are well integrated into the human resource strategy of a setting or organisation and accepted as the norm, they are likely to be well implemented and effective. Essentially, the time horizon of the evaluation should be long enough to catch all relevant differences in the consequences and costs of the alternatives compared [70]. Another limitation is the seasonal bias if a physical activity programme lasts less than one year. Levels of physical activity vary with seasonality and the subsequent effect of bad or extreme weather has been identified as a barrier to participation in physical exercise among various populations [84]. Weather accounts for nearly half of variance in measured physical activity programmes [85], and, therefore, in the effectiveness because of a better or worse participation rate and compliance. The pattern of seasonality also affects the kind of physical activity. Unusually hot, cold or wet weather conditions during the conduct of a programme should be appropriately be taken into account in the evaluation to ensure the generalisability of the results of the intervention. This holds especially for programmes of short duration. Also, seasonal effects could affect the sickness rate of people; in general, people have increased risks of getting ill in wintertime. Proper *et al.* measured sick leave days from work as an outcome of their exercise intervention. This intervention took place from May 2000 to January 2001 and left out the time from February to April with an above-average susceptibility particularly for airway diseases. Therefore, the seasonal effect could affect the costs and effectiveness of this intervention [41].

Extending the length of the follow-up time seems to be associated with a positive clinical and cost outcome impact of the intervention [37,48–50,53,86]. Such outcomes underscore the necessity of long-term support to sustain short-term changes in risk factors. Some studies included multiple sequential follow-up assessments in different time horizons [37,42,47,51]. These studies help to evaluate the extent to which early intervention effects endure over time.

Contrary to the clear recommendations in most guidelines, some studies neither discounted future costs nor effects even in cases of rather long time horizons, *i.e.*, up to 12 years [37].

In primary prevention, the promotion of physical activity is directed towards inactive people. It is a challenge to motivate these people to do physical exercise, particularly when they feel healthy. A person who likes sport is easy to enthuse for a new kind of physical activity. Therefore, the success of a primary prevention physical activity intervention mainly depends on the recruitment and response rate of the appropriate study population.

Although all studies measured the programme costs, decision-makers confronted with the question of whether or not to transfer and implement the programme need to be fully informed about the cost items included in the total programme costs. Even if most studies did provide a detailed description of the costs of the intervention programme in their country currency, data on the underlying quantities of resources used and the price year were often not displayed in the publications, thus making transferability of economic results difficult. In addition, outcomes of physical activity interventions were selected in accordance to the respective intervention aim. Yet, for direct comparisons of the different types of physical activity interventions similar outcomes would be important. For example, only three studies used QALYs as an outcome variable, the other studies could not be compared regarding their outcomes. Main areas of uncertainty were often not considered in the studies. The sensitivity analyses were often of low quality, e.g., insufficient explanation was given for the range of parameters chosen for the sensitivity analysis, leaving the impression of arbitrariness.

## 5. Conclusions

This review targets clinicians, behavioural scientists, researchers working in the field of public health, and decision-makers. It may, to some degree, show the difficulties of economic evaluation in the area of primary prevention. It aims to provide useful information for decision-makers, asking which study has the best methodological quality and which intervention can be transferred to another country. In sum, most of the studies reviewed conclude that the investigated programme provides good value for money compared with alternatives. However, it is not possible to compare these results directly. Some of the studies indicate that different approaches for increasing physical activity levels are advisable in different segments of the adult population.

The review has shown the differences in economic evaluation methods including costing, outcomes and results and that the overall quality of the economic evaluations varied widely across and within each category. Though several studies proved high economic and clinical evidence, the results of most of them are not transferable to a different country or setting without limitations. The main implication of this study is that high methodological quality and explicit reporting are important to assess potential generalisability or transferability of the results of the economic evaluations of physical exercise interventions. Authors of studies should explore through sensitivity analysis whether their results would apply in a different patient population or a different healthcare setting. The transferability can only be explored in detail if future studies comply more closely with the guidelines and recommendations for methodological standards of economic evaluations [32,35,87]. Hence, a considerable reduction in the variability of methods used in the evaluation of primary prevention programmes could be achieved if the authors reported the necessary data, like country-specific prevalence/incidence data, prices, years, physical units, treatment patterns, perspectives, and characteristics of settings and populations. Therefore, studies should ensure that all costs and outcomes are included and that resources are described and valued appropriately. This will enhance the comparability and generalisability of the outcomes and cost estimates of an intervention programme as transferability of economic evaluations is of growing importance to decision-makers due to scarce resources in the healthcare system.

## Figures and Tables

**Table 1.** Study characteristics and key findings.

Author (year published) [Ref.]	Study design/Type of EconA	Type of physical exercise intervention/alternative/length of intervention	Outcomes	Study Population: n/age (range or mean)/exclusion and/or inclusion criteria	Country/setting/year of study	Economic key findings	EURO conversion (2008)	Clin.*/ econ. evidence (h, l, m)**
Dzator *et al.* (2004) [51]	RCT/CEA	Self-directed intervention of PA and nutrition delivered by mail (low level) or by mail and group sessions (high level)/no intervention/16 weeks	Change in BMI, total and HDL cholesterol, blood pressure, PA (W/kg), nutrition fat intake	137 couples/all ages/IN: cohabitation for the first time, living together for < 2 years, no pregnancy for length of study/EX: CHD, severe asthma, diabetes	Australia/ home/ n.s.	1-year follow up: Average incremental costs/unit change in outcome variables:1) high intervention: AUD460; 2) low intervention: AUD459; 3) control: AUD462	No year of intervention	1+/m
Elley *et al.* (2004) [38]	Cluster RCT/CEA	Green Prescription: verbal and written exercise advice by GP and telephone exercise specialist/usual care/1 year	Total energy expended (change in PA), QALY	878/40-79 years/IN: less active (<2.5 hours of moderate activity per week)	New Zealand/ GPP/ 2000–2002	1) Monthly CER: NZD11/kcal/kg/day; 2) ICER: NZD1,756/ converted sedentary adult to an active state in 12 months	1) €8; 2) €1,268	1−/h
Finkelstein *et al.* (2002) [40]	RCT/CEA	WISEWOMAN Project: screening and counselling (e.g., walking, dance, chair-aerobics, weight training)/MI *vs.* EI/1 year	Risk of CHD, LYG	1586 women/40–64 years/IN: uninsured or underinsured with low annual income/take part in NBCCED-programme	USA/ community and healthcare sites/1996	1) IC of EI per person: USD191; 2) ICER: USD637/ 1%point additional decrease in 10 year probability of CHD for EI compared with MI; 3) nearly USD5,000/ LYG (n.sig.)	Apy (1996): 1) €226; 2) ICER: €753; 3) €5,911	1−/m
Robertson *et al.* (2001a) [50]	RCT/CEA	Otago: Individually home-based PA by district nurse/usual care/1 year	Falls and injuries	240/≥75 years/invited by GP/EX: abasia, receiving physiotherapy	New Zealand/ GPP/ 1998	1) ICER: NZD1,803/ fall prevented; 2) NZD7,471/ injurious fall prevented (cost saving for people older than 80 years)	1) €1,423; 2) €5,898	1+/h
Robertson *et al.*(2001b)[49]	CT/CEA	Otago: Individually home-based PA by general practicenurse/usualcare/1 year	Falls and injuries	450/≥80 years/invited by GP/EX: abasia, receiving physiotherapy	New Zealand/ GPP/ 1998	1) ICER: NZD1,519/ fall prevented;2) NZD3,404/ injurious fall prevented	1) €1,202;2)€2,694;	2+/h
Robertson *et al.* (2001c) [48]	RCT/CEA	Otago: Individually home-based PA by physiotherapist/usual care/2 years	Falls and injuries	233 women/≥80 years/invited by GP/EX: abasia, receiving physiotherapy	New Zealand/ GPP, home/ 1995–1997	1) ICER: NZD314/ fall prevented (1 year); NZD265/ fall prevented (2 years) 2) NZD457/ injurious fall prevented (1year); NZD 426/ injurious fall prevented (2 years)	1) €261; €220 2) €379; €353	1+/h
Stevens *et al.* (1998) [42]	RCT/CEA	Individual PA by exercise development officer/ EI vs MI/10 weeks	PA, number of sedentary people	714 participants/45–74 years/four subgroups (sedentary, low/high intermediate, active)/2 GPP/EX: disabled, CHD	UK/GPP/n.s.	1) £623/ one sedentary person doing more PA; 2) £2,498/ moving someone who is active but below min level	No year of intervention	1+/m
The Writing Group (2001) [47]	RCT/costs, effects (CEA)	PA counselling with current recommended care/usual care/2 years	Cardio-respiratory fitness, self-reported PA	874/35–75 years/IN: inactive, in primary care, in stable health, EX: chronic diseases, CHD	USA/GPP/ 1995–1997	1) For 2 years: IC/ participant of assistance intervention: USD500; 2) IC of counselling intervention/ participant: USD1,100	Apy (1996): 1) €591; 2) €1,300	1++/l
Proper *et al.* (2004) [41]	RCT/CBA, CEA	Worksite PA counselling/EI *vs.* MI/9 months	Sick leave, PA, cardiovascular fitness	299/44 years/IN: civil servants from three municipal services, performing office work at least 24 hours a week	Netherlands/ municipal services/ 2000–2001	CER without (with) imputation of effect data: 1) €5 (€3)/ extra energy expenditure (kcal/day); 2) €235 (€46)/ beat per minute of decrease in submaximale heart rate; 3) total net costs (9 months): €305; 4) benefits from sick leave reduction (1 year later): €635	Apy (2000): 1) €6 (€3); 2) €267 (€52); 3) €346; 4) €721	1+/m
Shepard (1992) [37]	CT/CBA, CEA	Employee fitness programme (rhythmic, aerobic type activity, stretching, cardio-respiratory activity)/no intervention/12 years	PA, absenteeism, corporate commitment	534/age n.s./office workers of two major insurance companies	Canada/ company/ 1977–1990	1) Programme benefits/worker/year (participation rate of 20%): CAD679; 2) ROI: CAD7; 3) Cost-benefit: CAD5 to 1	1) €757; 2) €8; 3) €5 to 1	2-/l
Chen *et al.* (2008) [36]	Cluster RCT/CUA	Walking/no intervention/12 weeks	Health service utilisation, QALY	98/>65 years	Taiwan/community/n.s.	ICER: USD15,103/ QALY gained	No year of intervention	1−/m
Dalziel/ Segal (2006) [44]	Cluster RCT/CUA (Markov Model)	Green Prescription: verbal and written exercise advice by GP and telephone exercise specialist/usual care/1 year	Lifestyle change, activity change, QALYs	878/40–79 years/42 GPP/IN: less active	New Zealand/ GPP/ 2000–2002	ICER: NZD2,053/ QALY gained (lifetime)	€1,483	1−/m
Lindgren *et al.* (2003) [43]	RCT/CEA, CUA (Markov Model)	Dietary advice by dietician and exercise instructions by physician/usual care/18 months	Physiological factors, QALYs, LYG	813 men/60 years/EX: CHD, diabetes, severe illness, no cholesterol, regular use of drugs	Sweden/ community/ 1992	ICER (declining effect of intervention): 1) Diet: SEK127,065/ LYG (SEK130,505/ QALY gained); 2) Exercise: SEK180,470/ LYG (SEK191,750/ QALY gained); 3) Exercise+diet: SEK201,375/ LYG (SEK201,375/ QALY gained)	1) €15,274 (€15,687); 2) €21,693 (€23,049); 3) €24,206 (€24,206)	1+/h
Munro *et al.* (2004) [39]	Cluster RCT/CUA	Free exercise classes (e.g., bowling, swimming, country walking, and tea dances) by qualified exercise leader/usual care/2 years	Mortality, hospital service use, health status, QALY	6420/>65 years/EX: PA score in the top 20%, patients who were unsuitable for exercise	UK/ community/ 2003–2004	ICER: €17,172/ QALY gained	ICER: €18,364	1−/h
Sims *et al.* (2004) [45]	Cluster RCT/CEA, CUA (Model)	Active Script Programme (ASP): training and support of GPs who deliver advice on PA/usual care/2 years	Number of advising GPs, patients becoming active or accruing health benefit, DALYs/deaths averted	670 GPs/practice population/20–75 years, sedentary	Australia/ GPP/ community/ 1999–2000, 2000–2001	1) AUD69/ patient to become more active (short term); 2) AUD138/ patient to accrue a health benefit; 3) AUD3,647/ DALY saved; 4) AUD48,924/ premature death averted	1) €62; 2) €123; 3) €3,258; 4) €48,708	1−/h

* (1++) High quality meta-analyses, systematic reviews of RCTs, or RCTs with a very low risk of bias; (1+) High quality meta-analyses, systematic reviews of RCTs, or RCTs with a low risk of bias; (1–) High quality meta-analyses, systematic reviews of RCTs, or RCTs with a high risk of bias; (2+) Well-conducted case-control, before-after studies or cohort studies with a low risk of confounding, bias or change and a moderate probability that the relationship is causal. (2–) Case-control, before-after studies or cohort studies with a high risk of confounding, bias or change and a high risk that the relationship is not causal. [28]

** h = high; l = low; m = moderate. *Abbreviations*: ASP: Active Script Programme; apy: Assumed price year; BMI: Body-mass-index; CHD: Cardiovascular heart disease; CG: Control Group; CT: Controlled trial; CBA: Cost benefit analysis; CE: Cost-effectiveness; CEA: Cost-effectiveness analysis; CER: Cost-effectiveness ratio; CUA: Cost utility analysis; DALY: Disability adjusted life years; EconA: Economic analysis; EI: Enhanced intervention; EX: Exclusion criteria; GP: General practitioner; GPP: General practitioner practices; HDL: High Density Lipoprotein; IN: Inclusion criteria; ICER: Incremental cost-effectiveness ratio; IG: Intervention Group; kcal: Kilocalorie; LYG: Life years gained; MI: Minimum intervention; NBCCED: National Breast and Cervical Cancer Early Detection; NB: Net benefit; n.s.: not stated; PA: Physical activity; QALY: Quality adjusted life year; RCT: Randomised controlled trial; ROI: Return on investment; SEK: Swedish Krona; W/kg: Watt/kilogram.

## Appendix

**Table 2.** Evaluation of economic evidence according to CHEC-List by Evers *et al.* (2005)^1^.

**Author's name (year)**	Is the study clearly described?	Are competing alternatives clearly described?	Is a well-defined research question posed in answerable form?	Is the economic study design appropriate to the stated objective?	Is the chosen time horizon appropriate in order to include relevant costs and consequences?^2^	Is the actual perspective chosen appropriate?	Are all important and relevant costs for each alternative identified?	Are all costs measured appropriately in physical units?	Are costs valued appropriately?	Are all important and relevant outcomes for each alternative identified?	Are all outcomes measured appropriately?	Are outcomes valued appropriately`?	Is an incremental analysis of costs and outcomes for each alternative performed?	Are all future costs and outcomes discounted appropriately?^3^	Are all important variables, whose values are uncertain, appropriately subjected to sensitivity analysis?	Do the conclusions follow from the data reported?	Does the study discuss the generalisability of the results to other settings and patient/ client groups?	Does the article indicate that there is no potential conflict of study researcher(s) and funder(s)?	Are ethical and distributional issues discussed appropriately?	Economic evidence
**Dzator*****et al.*****(2004) [51]**	0,5	1,0	1,0	1,0	0,5	0,0	0,5	0,0	0,5	1,0	1,0	1,0	1,0	1,0	1,0	1,0	1,0	1,0	0,0	**0,74**
**Elley *et al.* (2004) [38]**	1,0	0,5	1,0	1,0	0,5	1,0	1,0	1,0	1,0	1,0	1,0	1,0	1,0	1,0	1,0	1,0	0,5	1,0	0,0	**0,87**
**Finkelstein *et al.* (2002) [40]**	1,0	0,5	1,0	1,0	0,5	0,0	0,5	0,0	0,5	1,0	0,5	1,0	1,0	1,0	1,0	1,0	0,5	1,0	1,0	**0,74**
**Robertson *et al.* (2001a) [50]**	1,0	1,0	1,0	1,0	0,5	1,0	1,0	1,0	1,0	1,0	1,0	1,0	1,0	1,0	1,0	1,0	1,0	1,0	0,0	**0,92**
**Robertson *et al.* (2001b) [49]**	1,0	1,0	1,0	1,0	0,5	1,0	1,0	1,0	1,0	1,0	1,0	1,0	1,0	1,0	1,0	1,0	0,5	1,0	0,0	**0,89**
**Robertson *et al.* (2001c) [48]**	1,0	1,0	1,0	1,0	1,0	1,0	1,0	1,0	1,0	1,0	1,0	1,0	1,0	0,0	1,0	1,0	1,0	1,0	0,0	**0,89**
**Stevens *et al.* (1998) [42]**	1,0	1,0	1,0	0,5	0,0	0,0	0,0	0,0	0,0	1,0	1,0	1,0	0,0	1,0	1,0	1,0	1,0	1,0	0,0	**0,61**
**The Writing Group (2001) [47]**	1,0	1,0	0,5	0,0	1,0	0,0	0,0	0,0	0,0	1,0	1,0	1,0	1,0	0,0	0,0	0,5	0,0	1,0	0,0	**0,47**
**Proper *et al.* (2004) [41]**	1,0	1,0	0,5	1,0	0,0	0,5	1,0	0,0	0,5	1,0	1,0	0,5	1,0	1,0	1,0	1,0	1,0	1,0	0,0	**0,74**
**Shepard (1992) [37]**	0,5	0,5	0,5	1,0	1,0	0,0	0,5	0,5	0,5	1,0	0,5	0,5	0,0	0,0	0,0	1,0	0,5	0,0	1,0	**0,50**
**Chen *et al.* (2008) [36]**	1,0	1,0	1,0	1,0	0,0	0,0	1,0	0,0	0,5	1,0	1,0	1,0	1,0	1,0	0,0	1,0	0,5	1,0	1,0	**0,74**
**Dalziel K, Segal L (2006) [44]**	1,0	0,5	1,0	1,0	1,0	0,5	0,5	0,5	0,5	1,0	1,0	1,0	1,0	1,0	1,0	0,5	1,0	1,0	0,0	**0,79**
**Lindgren P *et al.* (2003) [43]**	1,0	1,0	1,0	1,0	1,0	1,0	0,5	0,5	0,5	1,0	1,0	1,0	1,0	1,0	1,0	0,5	0,5	1,0	0,0	**0,82**
**Munro *et al.* (2004) [39]**	1,0	1,0	1,0	1,0	1,0	0,5	1,0	1,0	1,0	1,0	1,0	1,0	1,0	0,0	1,0	1,0	0,5	1,0	0,0	**0,84**
**Sims J *et al.* (2004) [45]**	0,5	1,0	1,0	1,0	1,0	0,5	1,0	0,5	0,5	1,0	1,0	1,0	1,0	1,0	1,0	1,0	0,5	1,0	0,0	**0,82**

1 Asessment: Yes = 1; No = 0; Unclear= 0,5; Economic evidence grade: 0–0.50 = low; >0.50–0.80= moderate/limited; >0.80–1= high;

2 <1year = 0; 1–<2 years = 0.5; >2 years = 1;

3 The valuation of the reviewed studies without discounting was calculated with 1 if the evaluation period was less than 18 months and 0 if the time horizon was more than 18 months.

**Table 3.** Limitations of transferability of economic evaluation results according to Welte *et al.* (2004) ^1^ [35].

**Author (year)**	**Main Limitations with respect to transferability**
Dzator *et al.* (2004) [51]	duration of the intervention relatively short (16 weeks); higher economic status was over-represented in the study (potential bias); responders more motivated than non responders (selection bias); perspective not stated; only costs were discounted; price year not stated; high clinical and moderate economic evidence
Elley *et al.* (2004) [38]	control group may have taken part in exercise trial (potential bias); 1/3 of eligible participants did not participate (selection bias); large 95%CIs and imprecision around changes in major offset costs, especially healthcare utilisation costs and productivity costs (an overall cost-effectiveness from societal perspective could not be calculated); only costs were discounted; moderate clinical and high economic evidence
Finkelstein *et al.* (2002) [40]	baseline comparability of two groups not discussed; uninsured, low income women (US specific sample); no control group with no intervention; no discussion about women not taking part in interventions; no sensitivity analysis; perspective not stated; costs for single unit are not stated; price year not stated; only effects discounted; moderate clinical and moderate economic evidence
Robertson *et al.* (2001a) [50]	district nurse (potential instructor bias); only cost-saving for people older than 80 years; costs could be different in an urban area (e.g., less transport costs); high clinical and high economic evidence
Robertson *et al.* (2001b) [49]	general practice nurse (potential instructor bias); costs could be different in an urban area (e.g., less transport costs); no randomisation; moderate clinical and high economic evidence
Robertson *et al.* (2001c) [48]	research physiotherapist (potential instructor bias); costs could be different in an urban area (e.g., less transport costs); no discounting; high clinical and high economic evidence
Stevens *et al.* (1998) [42]	perspective not stated; no explanation for choice of comparator; no data on effectiveness; exercise development officer (potential therapist bias); short intervention time (10 weeks); unit costs could be halved with a better recruitment strategy; ICER not stated; perspective not stated; physical units not stated; no discount rate; price year not stated; valuation of the costs not mentioned; high clinical and moderate economic evidence
The Writing Group (2001) [47]	only effects are differentiated; not significantly effective for men; perspective not stated; discount rate not stated; physical units not stated; sensitivity analysis not stated; cost measurement and valuation not stated; high clinical and low economic evidence
Proper *et al.* (2004) [41]	large CIs of CE-Ratios (not statistical significant);health care costs only accountable by municipal service; underpowered trial= 94–167; potential other benefits excluded like employer turnover, productivity, commitment; CBA is a CEA (no monetary valuing of the benefits); price year not stated; physical units not stated; high clinical and moderate economic evidence
Shepard (1992) [37]	costs not stated explicitly; study years not stated appropriately; no randomisation of the study population; not all items of programme costs calculated; CEA and CBA are not explained adequately; no further description of the target population; ICER not stated; perspective not stated; no discounting; physical units not clearly stated; sensitivity analysis not stated; moderate clinical and low economic evidence
Chen *et al.* (2008) [36]	perspective not stated; physical units not stated; no sensitivity analyses; short time horizon; valuation of utilisation not stated; only programme costs included; high clinical and moderate economic evidence
Lindgren P *et al.* (2003) [43]	only 60 year old men - transferability to other ages unclear; Markov Model uses risk factors taken from Framingham (calculated for UK and Germany); only Human Capital approach; Physical activity programme not described; high clinical and high economic evidence
Dalziel K, Segal L (2006) [44]	based on many assumptions; wide range of ICER; short follow up period in the primary clinical trial (Elley *et al.*2004);moderate clinical and moderate to high economic evidence
Munro *et al.* (2004) [39]	SF-36 non responders were assumed having no health benefit; benefit by participants in exercise programmes greater than the suggests (potential selection bias); exclusion of the top 20% (selection bias); participation rate and levels of missing data are correlated; low recruitment rate; follow-up period too short for mortality and admission rates; no discounting; moderate clinical and high economic evidence
Sims J *et al.* (2004) [45]	method of discounting not explained; model and costs not described in detail; no indirect costs; percentage of patients to become active assumed; moderate clinical and high economic evidence

Abbreviations: CBA: Cost-benefit analysis; CE: Cost-effectiveness; CEA: Cost-effectiveness analysis; ICER: Incremental cost-effectiveness ratio; CI: Confidence interval; UK: United Kingdom; US: United States.
